# Water-Recyclable Chitosan-Based Ion-Imprinted Thermoresponsive Hydrogel for Rare Earth Metal Ions Accumulation

**DOI:** 10.3390/ijms231810542

**Published:** 2022-09-11

**Authors:** Yuheng Qiu, Kaiqi Ding, Liwen Tang, Ziyu Qin, Mengting Li, Xueqiong Yin

**Affiliations:** 1 Hainan Provincial Fine Chemical Engineering Research Center, Hainan University, Haikou 570228, China; 2 College of Chemical Engineering and Technology, Hainan University, Renmin Avenue 58th, Haikou 570228, China

**Keywords:** thermoresponsive hydrogel, La^3+^-imprinting, rare earth metal

## Abstract

The demand for rare earth metal increases rapidly in the modern high-tech industry and therefore the accumulation of rare earth metal ions from an aqueous environment becomes a significant concern worldwide. In this paper, a water-recyclable chitosan-based La^3+^-imprinted thermoresponsive hydrogel (CLIT) was prepared to accumulate La^3+^ from solution. The CLIT was characterized by DSC, FITR, Raman spectroscopy, XPS, and SEM, which revealed obvious reversible thermosensitivity and imprinted sites of La^3+^ ions. An adsorption capacity of 112.21 mg/g to La^3+^ ions was achieved on CLIT under its optimum adsorption conditions (pH 5, 50 °C, 60 min). The adsorption could be well illustrated by second-order kinetics and Freundlich isotherm models. The La^3+^-adsorbed CLIT could be recycled only by rinsing with 10 °C cold water, with a desorption rate of 96.72%. After ten cycles of adsorption-desorption, CLIT retained good adsorption capability. In the solution containing six ions, the adsorption coefficients k_La3+/Mn+_ of CLIT were 2.04–3.51 times that of non-imprinted hydrogel, with k_La3+/Y3+_, k_La3+/Gd3+_, k_La3+/Al3+_, k_La3+/Fe3+_ and k_La3+/Cu2+_ being 1.67, 2.04, 3.15, 2.72 and 4.84, respectively.

## 1. Introduction

With the rapid development of society and technology, rare earth elements are in increasing demand worldwide due to their more and more important roles in the high-tech industry, such as superconducting magnets, rechargeable batteries, military products, and laser material, etc. The accelerated mining, processing, and application of rare earth metals are accompanied by the introduction of large quantities of rare earth metal ions (REMs) into water resource systems [1]. The concentration of rare earth metal elements in natural lakes and rivers is usually required to be less than 20 µg/L [2,3]. On one hand, the releasing in mine drainage results in an unnecessary loss of rare earth resources; on the other hand, the accumulation of REMs in water can also have irreversible effects on living organisms, such as retarding the growth and development of plants and causing damage to the nervous system and connective tissues in animals and humans [4,5], etc. Therefore, the recovery of REMs from the environment is important for building a resource-conserving and environment-benign society.

Up to now, various methods have been investigated for separating different types of REMs from solution, such as the step-by-step precipitation method, liquid-liquid extraction, membrane separation, solid-liquid extraction, and adsorption [6,7,8,9,10]. Among these, the adsorption method is considered a promising alternative to conventional processes for the effective recovery of REMs, especially from low concentration solutions, due to its simple operation, high recovery efficiency, low cost, and wide applicability [11,12]. Various adsorbents, including SBA-15, graphene oxide (GO), clay, carbon materials, chitosan and its derivatives, β-cyclodextrin, silica and nanocomposites, have been widely used for REMs recovery [13,14,15,16,17,18]. However, rare earth elements are well-known for their difficulty to isolate owing to their similar hydrated ionic radii and properties with other metal elements [19]. Therefore, the adsorption selectivity appears to be important for REMs separation [20]. In order to improve the adsorption selectivity to metal ions, ion-imprinted polymers with the ability to recognize specific ions with the same shape and size as the template are widely investigated for selective separation of metal ions. Lanthanide ions (La^3+^) have also been used as the template ions to prepare ion-imprinted polymers for their adsorption separation. For example, Rohani et al. used La^3+^ as the template ions, with either a Schiff base or azobenzene as the complexing agents, and vinyl pyridine as the monomer to prepare La^3+^-imprinted polymers (La-IIP-Schiff, La-IIP-Azo). The maximum adsorption capacity of La-IIP-Schiff and La-IIP-Azo to La^3+^ at pH 6 was 25.0 mg/g and 24.3 mg/g, respectively [21]. Another La^3+^-imprinted polymer La-IIP-MAA/Fe_3_O_4_-GO was prepared by Shi et al., using magnetic GO as the carrier and methacrylic acid as the functional monomer. The maximum adsorption capacity of La-IIP-MAA/Fe_3_O_4_-GO could reach 110.98 mg/g at pH 6 and 35 °C. La-IIP-MAA/Fe_3_O_4_-GO also showed certain selectivity to La^3+^ in a mixed solution containing La^3+^, Ce^3+^, Gd^3+^, Y^3+^, Fe^3+^ and Al^3+^ [22]. Although ion imprinting technology has substantially improved the selectivity of the adsorbent, all existing La^3+^-imprinted materials still required acid like HCl and HNO_3_ to elute the metal ions and thus regenerate the material. In addition to the potential environmental pollution problems caused by acid, acid washing may also cause the structural change of the adsorbent such as polymer chain degradation, imprinting site disintegration, and subsequent decreasing adsorption performances [23]. Thermoresponsive hydrogels have attracted a lot of attention in stimulus-responsive hydrogel systems, which exhibit significant swell-shrink behavior when the temperature is below or above a temperature known as the volume phase transition temperature (VPTT) [24]. Lu et al. prepared thermosensitive Eu^3+^ imprinted membranes (Eu-IIMs) for separating Eu^3+^ from La^3+^, Gd^3+^ and Sm^3+^, which had been synergistically stacked by GO and modified silicon dioxide (kSiO_2_) and Ag nanoparticles, using Eu^3+^ ions as the templates and N-isopropylacrylamide (NIPAM) and acrylamide as the thermosensitive monomer. The Eu^3+^-rebinding capacity of Eu-IIMs was 101.14 mg/g and the adsorptive selectivity for Eu^3+^/La^3+^, Eu^3+^/Gd^3+^, Eu^3+^/Sm^3+^ was 1.82, 1.57, 1.45, respectively. However, the adsorption saturated Eu-IIMs should also be desorbed by 1.0 M HCl solution [25].

**Figure 1:**
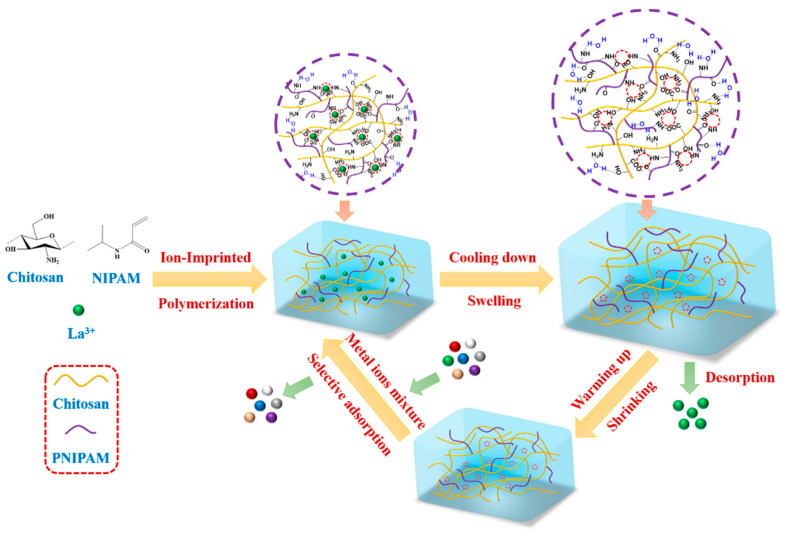
Preparation, adsorption and desorption process of CLIT.

To overcome the shortcoming of using acid to regenerate the ion-imprinted materials, a novel water-recyclable chitosan-based La^3+^-imprinted thermoresponsive hydrogel (CLIT) was presented in the current work. Here, chitosan was used as the matrix because chitosan is the second abundant polysaccharide having many unique features, such as being biodegradable, highly hydrophilic, easy to be modified, and having a good ability to adsorb metal ions, etc. [26] CLIT was prepared through a gradient heating ion-imprinting polymerization method (GHIP) proposed by our group, with La^3+^ as the template ion, chitosan as the matrix, and NIPAM as the thermosensitive monomer. CLIT can be easily regenerated by washing with cold water (about 10 °C), avoiding the adverse effects that the use of acids may have on the environment. The structure of CLIT was characterized by DSC, FTIR, Raman spectroscopy, SEM, and XPS. The adsorption capacity and adsorption selectivity with interfering ions (Y^3+^, Gd^3+^, Al^3+^, Fe^3+^ and Cu^2+^) were investigated. The adsorption kinetics was simulated with a quasi-first-order kinetic equation and quasi-second-order kinetic equation, and the adsorption isotherms were simulated with the Freundlich isotherm model and Langmuir isotherm model. Moreover, the desorption performance and the reusability were studied to understand the practical application potential of CLIT. The preparation, adsorption and desorption process of CLIT was schematically illustrated in Figure 1.

## 2. Results and Discussion

### 2.1. Characterization of CLIT

**Figure 2:**
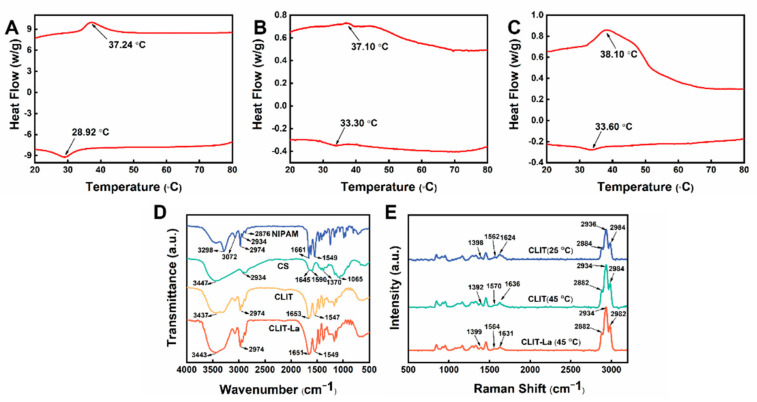
DSC curves of CLIT during repeated tests (the first test (**A**), second test (**B**), and third test (**C**)); FTIR spectra of CS, NIPAM, CLIT, CLIT-La (**D**); Raman spectra of hydrogels CLIT (25 °C), CLIT (45 °C), CLIT-La (45 °C) (**E**).

The preparation conditions of CLIT were optimized as elucidated in Table S1, Figures S1 and S2. DSC was used to study the temperature sensitivity of CLIT, which was measured three times during continuous heating and cooling in the range of 10–90 °C. Figure 2A–C showed the results of the first, second and third DSC measurement of CLIT, respectively. The VPTT of the heating stage and cooling stage were 37.24 °C and 28.92 °C in the first test, 37.10 °C and 33.30 °C in the second test, and 38.10 °C and 33.60 °C in the third test, respectively. After three cycles of DSC tests, the VPTT during heating had no obvious change. The VPTT during cooling increased a little after the first processing, and almost no change after the second processing. The results indicated that CLIT had good reversible phase change capability. The structure became more stable after shrinking and swelling during heating and cooling. The results provided the basis for the subsequent recycling and potential application for La^3+^ adsorption.

CLIT was used to adsorb La^3+^ ions at 50 °C and was then freeze-dried directly, getting the material containing La^3+^ (CLIT-La). The spectra of CLIT-La, freeze-dried CLIT, NIPAM and CS were measured with FTIR (shown in Figure 2). As known from Figure 2D, in the FTIR spectra of NIPAM, the absorption at 3298 cm^−1^ and 3072 cm^−1^ were the peaks of the stretching vibration and the multiplicity of the bending vibration of N-H, respectively. The peaks of the C-H stretching vibration of -CH_3_, -CH_2_- and CH in NIPAM appeared at 2974, 2934 and 2876 cm^−1^, respectively [27]. The strong adsorption peak at 1661 cm^−1^ was the C=O stretching vibration peak of the amide group, and the N-H bending vibration superimposed with the C-N stretching vibration showed an absorbance at 1549 cm^−1^ [28]. The strong absorption peaks at 3447 cm^−1^ and 2934 cm^−1^ in the CS spectrum were the N-H and O-H stretching vibration peak and C-H absorption peak, respectively [29]. The symmetrical C-O-C stretching vibration peak appeared at 1065 cm^−1^. The stretching vibrational peaks of C=O (amide band I), N-H (amide band II) and C-N (amide band III) were found at 1645, 1590 and 1370 cm^−1^, respectively [30].

In the spectrum of CLIT, the C-H characteristic peaks of NIPAM around 2900 cm^−1^ could be observed clearly. The C=O stretching vibration peak from NIPAM and CS, and the deformation vibration peak of N-H, showed up at 1653 cm^−1^ and 1547 cm^−1^, respectively. The FTIR spectrum of CLIT demonstrated that NIPAM had been successfully polymerized onto CS, giving the thermosensitive property to CLIT. Comparing CLIT with CLIT-La, the peak of C=O, N-H at 1652 cm^−1^, 1547 cm^−1^ in the spectrum of CLIT shifted to 1651 cm^−1^, 1549 cm^−1^ in CLIT-La respectively [31]. Moreover, the O-H and N-H stretching absorption of CLIT at 3437 cm^−1^ shifted to 3443 cm^−1^ in CLIT-La [32]. Above peak shifts demonstrated that C=O, N-H and O-H groups are important to the La^3+^ adsorbing process, and revealed that there was chemical coordination between the CLIT and La^3+^ ions.

The hydrogel CLIT was measured at 25 °C (CLIT (25 °C)) and 45 °C (CLIT (45 °C)), and the CLIT-La hydrogel was measured at 45 °C (CLIT-La (45 °C)) by Raman spectroscope, respectively. The Raman spectroscopy of CLIT (25 °C), CLIT (45 °C), CLIT-La (45 °C) is shown in Figure 2E. The peaks at approximately 1500–1700 cm^−1^ represented the hydrophilic groups (C=O and N-H), while the peaks at about 2800–3000 cm^−1^ represented the hydrophobic groups (CH_3_, CH_2_ and CH) [33]. As the test temperature rose from 25 °C to 45 °C, the N-H vibrational peak in CLIT moved from 1562 cm^−1^ to 1570 cm^−1^. Moreover, the C=O vibrational peak shifted from 1624 cm^−1^ to 1636 cm^−1^. These differences are probably attributed to the weakening or even loss of hydrogen bonding forces between N-H---O-H or C=O---H-O with increasing temperature [34,35]. The CH_3_ stretching vibration peak located at 2984 cm^−1^ had no change. However, the absorption peaks corresponding to CH and CH_2_ both changed after heating. The stretching vibration peak of CH showed a change from 2884 cm^−1^ to 2882 cm^−1^ with temperature increasing. At the same time, the stretching vibration peak of CH_2_ shifted from 2936 cm^−1^ to 2934 cm^−1^. These results indicated that the hydrophobic interaction between CH and CH_2_ was the main force for the phase transition of CLIT. The Raman results further suggested that CLIT was thermosensitive and the isopropyl groups in NIPAM played a key role in the phase transition change [33].

The Raman spectra of CLIT and CLIT-La were compared at 45 °C. After adsorption of La^3+^, the peak of C=O shifted from 1636 cm^−1^ to 1631 cm^−1^ and the C-O vibrational peak of CH_2_OH changed from 1392 cm^−1^ to 1399 cm^−1^, and the peak of N-H shifted from 1570 cm^−1^ to 1564 cm^−1^. The shifts of the three peaks were attributed to the generation of chemical bonds between La^3+^ and C=O, -O-H and the surrounding N-H groups [36]. The C-H peak at 2984 cm^−1^ shifted to 2982 cm^−1^, which might be due to the coordination weakening the adjacent hydrophobic interactions. Theoretically, when a La^3+^-containing CLIT is washed with low-temperature deionized water, the morphology of the adsorbent will be changed by the swelling effect of the thermosensitive hydrogel. As a result, the affinity of the imprinting sites will be weakened [37]. Therefore, the coordination interactions between CLIT and La^3+^ become weaker and even break. Then the La^3+^ ions are released and flow away with water. As a result, the adsorbent is recycled and La^3+^ ions are separated and concentrated.

**Figure 3:**
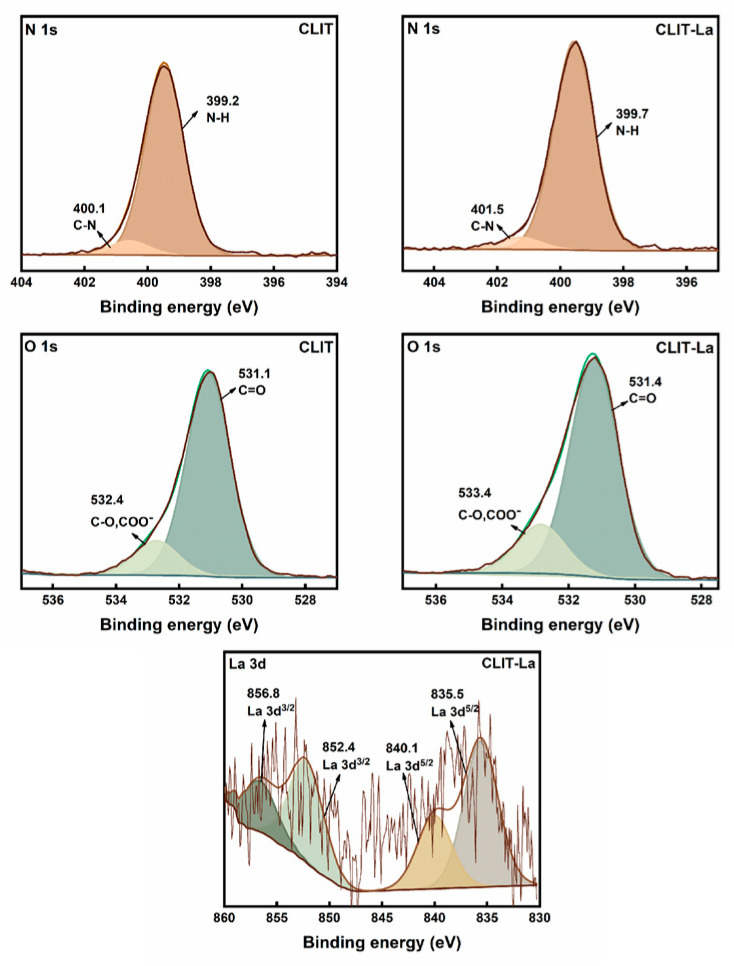
XPS curves of CLIT and CLIT-La.

The XPS measurement was also used to measure the structure of CLIT and CLIT-La. The N 1s, O 1s and La 3d spectra of CLIT and CLIT-La are shown in Figure 3. In addition, we give the binding energy of CLIT and CLIT-La in Table 1. After the adsorption of La^3+^ ions, the N1s peak of N-H and C-N bonds showed a shift from 399.2 eV to 399.7 eV and 400.1 eV to 401.5 eV [38]. CLIT-La expressed a higher binding energy than CLIT, which indicated that the amino and imine group in CLIT was involved in the adsorption of La^3+^ [39]. In the O 1s plot of CLIT, the peaks at 531.1 eV and 532.4 eV were corresponding to C=O and C-O (COO^−^), respectively. After adsorbing La^3+^, the C=O peak shifted from 531.1 eV to 531.4 eV, and the C-O (COO^−^) peak changed from 532.4 eV to 533.4 eV [40]. The results suggested that the C=O, C-O and COO^−^ were also related to the bonding between CLIT and La^3+^. Moreover, there were the La 3d^5/2^ characteristic peaks at 840.1 eV and 835.5 eV, and La 3d^3/2^ characteristic peaks at 856.8 eV and 852.4 eV, confirming that La^3+^ ions were successfully combined with CLIT [41].

**Figure 4:**
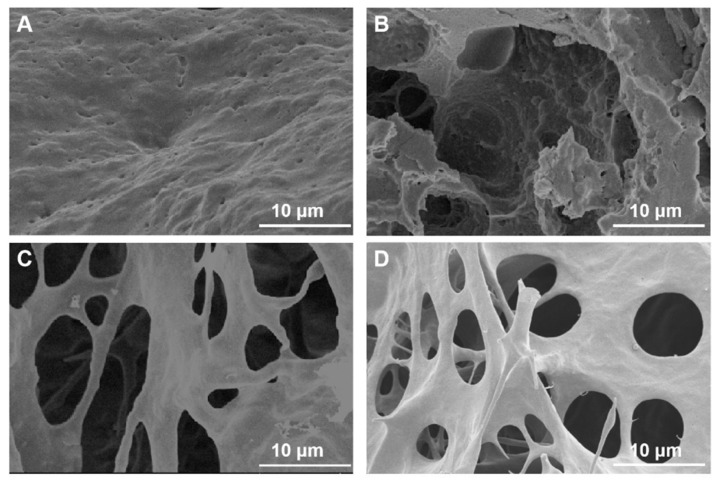
SEM images of CLIT-La ((**A**) surface; (**B**) cross section) and CLIT ((**C**) surface; (**D**) cross section).

CLIT-La and CLIT hydrogels were directly freeze-dried for 24 h before SEM testing. The SEM image in Figure 4A showed that the surface of CLIT-La was dense and coarse with some tiny holes. In the cross-sectional view of CLIT-La (Figure 4B), there were distinct pits and a porous structure inside the adsorbent, while the solid portion was also dense with tiny holes, similar to the surface. Figure 4C was the surface image of CLIT after removing La^3+^ with 10 °C water. There were obvious pores on the surface of CLIT. The cross-sectional image of CLIT (Figure 4D) looked similar with that of the surface image in Figure 4C. The SEM differences of CLIT and CLIT-La expressed that CLIT had a more loose and porous structure than CLIT-La. This was due to the thermosensitive CLIT swelling during rinsing with low temperature water for desorption of La^3+^. At low temperature, I the hydrogen bonds between water and CLIT increase, which causes more water staying in CLIT hydrogel. After freeze-drying, the water molecules sublimed and then left more pores in CLIT. Furthermore, the coordination bonds between La^3+^ and CLIT broke due to hydrogel swelling, and then the La^3+^ ions were eluted by water, with the empty sites remaining after freeze-drying. The SEM images further confirmed that the structure of CLIT altered with temperature and the imprinted La^3+^ could be removed by rinsing with low temperature water. In other words, the CLIT hydrogel is thermoresponsive and water-recyclable.

### 2.2. Adsorption Performance of CLIT

#### 2.2.1. Effects of Adsorption Temperature

**Figure 5:**
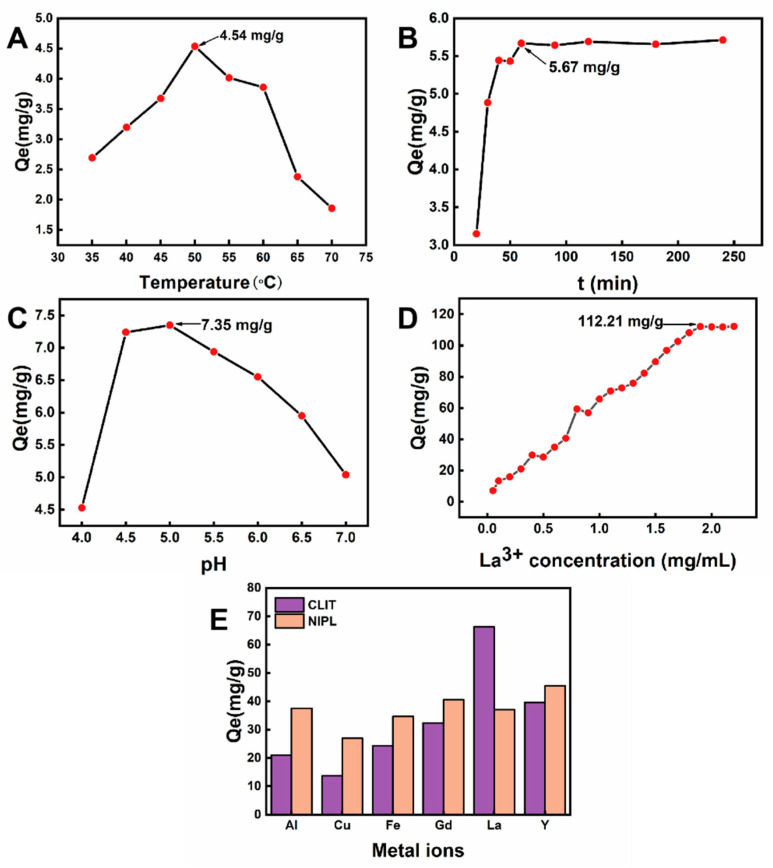
The effects of temperature (**A**), adsorption time (**B**), the pH value of the adsorption solution (**C**), the initial La^3+^ concentration (**D**) on the adsorption capacity of CLIT; (**E**) The adsorption capacity of CLIT and NIPL to metal ions coexisting in the same solution (containing Al^3+^, Cu^2+^, Fe^3+^, Gd^3+^, La^3+^ and Y^3+^).

From the DSC test results, it was clear that the VPTT of CLIT was about 38 °C during the heating process. Therefore, the effects of adsorption temperature of CLIT were explored in the range of 35–70 °C. CLIT hydrogel (dry weight of 0.1 g) was placed in 20 mL of La^3+^ solution (pH = 5.7, 50 μg/mL). After adsorption for 1 h, the concentration of La^3+^ in the filtrate was measured. As shown in Figure 5A, the maximum adsorption capacity of CLIT was 4.54 mg/g at 50 °C. At temperatures below 50 °C, the adsorption capacity of La^3+^ increased with temperature increasing. At temperatures above 50 °C, the amount of La^3+^ adsorbed by CLIT decreased with further increases the temperature. At 35 °C (<VPTT), the pores of the CLIT were too large to retain La^3+^ ions from the adsorption solution because CLIT was in a complete swelling state. As the temperature rose, some hydrogen bonds between CLIT and water molecules (such as C=O---H-O, H-N---H-O, N-H---OH_2_ from PNIPAM, O--H-O, N---H-O, O---H–N from chitosan) were broken [42,43]. The hydrogel lost some water and shrunk, then gradually restored the imprinting sites of La^3+^ and therefore the adsorption capacity increased. However, when the temperature was too high, the hydrogen bond network disintegrated further and the hydrogel shrank too much, making it impossible for La^3+^ ions to enter and thus the adsorption capacity gradually decreased.

#### 2.2.2. Effects of Adsorption Time

CLIT hydrogel (dry weight of 0.1 g) was placed in 20 mL of La^3+^ solution (pH = 5.7, 50 μg/mL). The adsorption was carried out at 50 °C for 20 min, 30 min, 40 min, 50 min, 60 min, 1.5 h, 2 h, 2.5 h, 3 h and 4 h to measure the effects of adsorption time on the adsorption capacity. As shown in Figure 5B, the adsorption capacity of CLIT on La^3+^ increased gradually with the increase of adsorption time from 20 min to 60 min. The adsorption equilibrium was almost reached at 60 min with an equilibrium adsorption capacity of 5.67 mg/g. Upon increasing the adsorption time further after 60 min, the adsorption amount did not change significantly. The results indicated that 60 min was the optimum time to reach adsorption equilibrium.

#### 2.2.3. Effects of pH Value of La^3+^ Solution

The pH value of La^3+^ solution (20 mL, 50 μg/mL) was adjusted to 4.0, 4.5, 5.0, 5.5, 6.0, 6.5 and 7.0 using sodium hydroxide (0.1 mol/L) and nitric acid (0.1 mol/L) before adding the CLIT hydrogel (dry weight of 0.1 g). The adsorption was carried out at 50 °C for 60 min. The adsorption capacities at different pH values are shown in Figure 5C. The adsorption capacity of CLIT to La^3+^ first increased with the increase of the initial pH of the La^3+^ solution, and reached a maximum value of 7.35 mg/g at pH = 5. As the pH further increased, the adsorption capacity of La^3+^ decreased gradually. When the pH was 4, there were more protons in the solution, which competed with La^3+^ to bind to CLIT and form protonated groups, such as NH_2_^+^, NH_3_^+^, OH_2_^+^, -C=OH^+^ [44]. The repulsion from the protonated groups weakened the attachment of La^3+^ to CLIT [39]. Therefore, the adsorption amount of CLIT to La^3+^ was low in the low pH solution. Moreover, in the La^3+^ solution with a too high pH value, the hydrolysis of La^3+^ might form less soluble La compounds [45], resulting in a lower adsorption amount.

#### 2.2.4. Effects of Initial La^3+^ Concentration

Twenty mL of La^3+^ solution with different initial concentration (0.05–2.2 mg/mL) was put into a 50 mL beaker. The CLIT hydrogel (dry weight of 0.1 g) was added to the La^3+^ solution whose pH had been previously adjusted to 5.0. Then the adsorption was carried out for 60 min at 50 °C. The amount of La^3+^ adsorbed at different initial La^3+^ concentration was shown in Figure 5D. Before the initial concentration of La^3+^ ions increased to 1.9 mg/mL, the adsorption amount of La^3+^ by CLIT increased continually. The adsorption amount increased from 7.11 mg/g at La^3+^ concentration of 0.05 mg/mL to 112.21 mg/g at 1.9 mg/mL. When the initial La^3+^ concentration continued to increase higher than 1.9 mg/mL, the adsorption amount had no further change, and the expression of the equilibrium was achieved at 1.9 mg/mL.

### 2.3. Adsorption Selectivity of CLIT

The corresponding La^3+^ non-imprinted hydrogel (NIPL) was prepared according to the same preparation protocol as CLIT in the absence of the template La^3+^ ions. NIPL and CLIT were put into a solution containing six metal ions (including La^3+^, Al^3+^, Cu^2+^, Fe^3+^, Gd^3+^, and Y^3+^) to test the adsorption selectivity of CLIT, respectively. The adsorption capacities and adsorption coefficients of each ion by CLIT and NIPL were calculated and shown in Figure 5E and Table 2.

From Figure 5E, it could be seen that the adsorption capacity of CLIT to La^3+^ was 66.32 mg/g, less than the adsorption capacity of CLIT in the solution without the interfering ions (112.21 mg/g). The adsorption amounts of other metal ions were 21.03 mg/g, Cu^2+^ 13.69 mg/g, Fe^3+^ 24.34 mg/g, Gd^3+^ 32.44 mg/g, Y^3+^ 39.62 mg/g. The adsorption capacity to rare earth meal ions Gd^3+^ and Y^3+^ was higher than that to Al^3+^ and the transition metal ions (Cu^2+^ and Fe^3+^). The adsorption amount of La^3+^ by NIPL was 37.11 mg/g. Moreover, the adsorption amounts of other metal ions were Al^3+^ 37.54 mg/g, Cu^2+^ 26.98 mg/g, Fe^3+^ 34.72 mg/g, Gd^3+^ 40.56 mg/g, and Y^3+^ 45.49 mg/g, respectively. For NIPL, there were no obvious adsorption differences between La^3+^ and the other metal ions in the solution, whereas NIPL even had an higher adsorption capacity to Gd^3+^ and Y^3+^ than La^3+^.

The selective adsorption coefficients of CLIT in the solution with six metal ions were k_La3+/Al3+_ 3.61, k_La3+/Cu2+_ 5.66, k_La3+/Fe3+_ 3.09, k_La3+/Gd3+_ 2.27, and k_La3+/Y3+_ 1.83, respectively, while, the adsorption coefficients of NIPL were k_La3+/Al3+_ 0.99, k_La3+/Cu2+_ 1.42, k_La3+/Fe3+_ 1.08, k_La3+/Gd3+_ 0.91, and k_La3+/Y3+_ 0.80, respectively. The adsorption coefficients of CLIT were 2.04–3.51 times that of NIPL. CLIT expressed much better selective adsorption ability to La^3+^ than NIPL, which proved that the La^3+^-imprinted CLIT had specific recognition sites of the template La^3+^ ions and therefore the adsorption coefficients of CLIT were much larger than those of NIPL. The adsorption performance of CLIT and NIPL revealed that the ion imprinting technique did improve the adsorption selectivity of the chitosan-based hydrogel to rare earth metal ions. Since the cavities in ion-imprinted polymers have a shape and size consistent with the template ions, the ions larger or smaller in size than the imprinted template are relatively incapable of being intercepted [46]. The ions Cu^2+^, Al^3+^, Fe^3+^ have a much smaller ionic radius (53–72 pm) than La^3+^ (106 pm), while other rare earth metal ions Y^3+^ and Gd^3+^ had a slightly smaller ionic radius (90–93.8 pm) with La^3+^. Therefore, CLIT had the highest adsorption capacity to La^3+^ and expressed higher adsorption capacity to Y^3+^, Gd^3+^ than to Cu^2+^, Al^3+^, and Fe^3+^.

### 2.4. Adsorption Mechanism of CLIT

#### 2.4.1. Kinetics Studies

**Figure 6:**
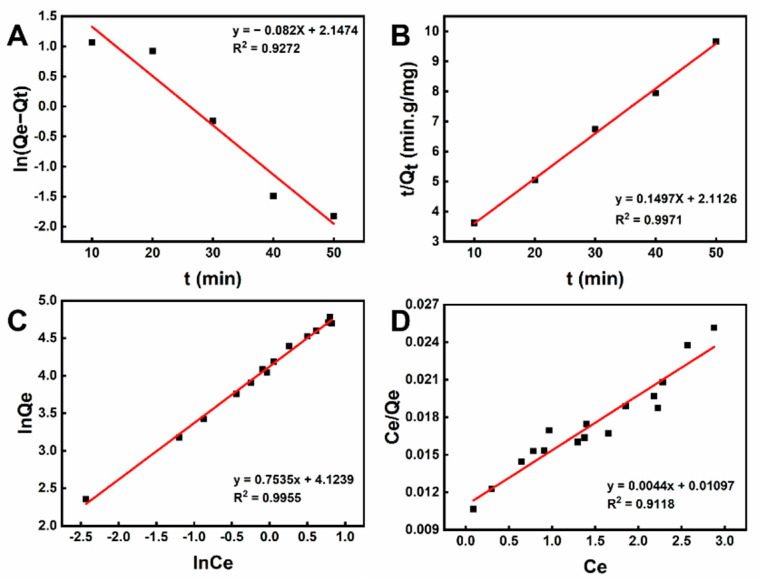
Adsorption simulation of CLIT with (**A**) quasi-first-order-kinetics; (**B**) quasi-second-order-kinetics; (**C**) Freundlich isotherm model; (**D**) Langmuir isotherm model.

The adsorption kinetic was studied through stimulation with quasi-first-order kinetic equations and quasi-second-order kinetic equations. The results of the simulations were shown in Figure 6A,B. The adsorption kinetic results indicated that the adsorption of CLIT to La^3+^ was more in line with the second-order kinetics (R^2^ = 0.9971) than the first-order equation (R^2^ = 0.9272,), which revealed that chemisorption played an important role in the adsorption of La^3+^ by CLIT [47,48].

#### 2.4.2. Adsorption Isotherms

The Freundlich and Langmuir equations were used as the adsorption isotherm models to study the La^3+^ adsorption process. The results of the adsorption isotherm simulation were shown in Figure 6C,D and Table 3. Both Freundlich and Langmuir adsorption models had a high fit for CLIT adsorption to La^3+^ [49]. However, the Freundlich model with R_f_^2^ = 0.9955 had a better fit than the Langmuir model with R_l_^2^ = 0.9118. According to the ion-imprinting technology, there should be specific imprinting sites for the template ions, forming heterogeneous chemical and morphology structure on the surface of the hydrogel. The imprinting sites enabled the selective adsorption for the La^3+^ ions. However, since the interactions between the adsorbents and La^3+^ ions are complex, based on the simulation results with the empirical equations, it could be concluded that the adsorption is heterogeneous and happens to fit the Freundlich-type model. Moreover, 1/*n* < 1 in the Freundlich isothermal adsorption model indicated that the adsorption of La^3+^ by CLIT was easy to carry out [50].

### 2.5. Desorption Performance of CLIT

CLIT hydrogel (dry weight of 0.1 g) was used to adsorb La^3+^ in a solution containing 1.9 mg/mL La^3+^ at pH 5, 50 °C for 60 min. The La^3+^-adsorbed hydrogel was then desorbed and recycled by washing with cold water. The effects of temperature of washing water (5 °C, 10 °C, 15 °C, 20 °C, 25 °C and 30 °C), desorption time (5 min, 10 min, 15 min, 20 min, 25 min and 30 min), and repeated operation times of desorption (1, 2, 3, 4 and 5 times) on the desorption rate of CLIT were investigated.

**Figure 7:**
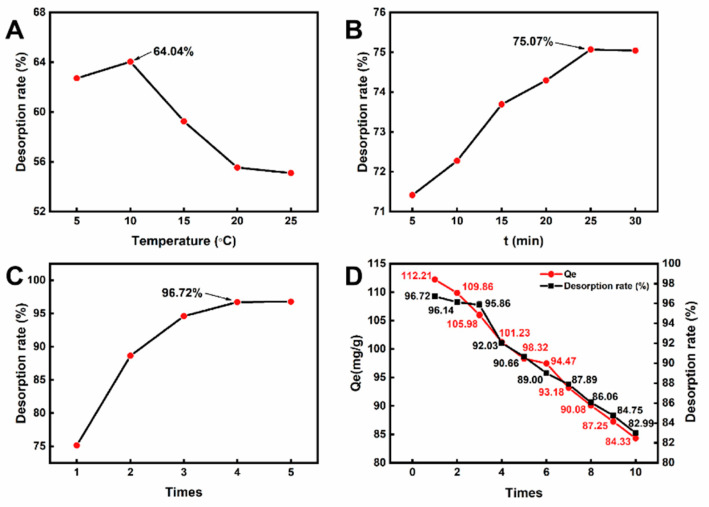
Effects of (**A**) eluent temperature, (**B**) soaking time, (**C**) repeated desorption times on CLIT desorption and the 10 cycle regeneration performance of CLIT (**D**).

The effects of eluent temperature on CLIT desorption for 10 min after one operation is shown in Figure 7A. At 5 °C, the desorption rate was lower than the maximum desorption rate obtained at 10 °C (64.04%). Further increasing the eluent temperature caused the desorption rate to decrease. The desorption rate decreased to 55.10% at 25 °C. Desorption is an endothermal reaction, and higher temperature is more beneficial for the desorption [51]. From a thermodynamics view, the desorption rate of CLIT should be higher at a higher temperature. However, to the thermosensitive hydrogel, the material shrinks more at a higher temperature [52], causing the pore size to decrease and making the adsorbed ions more difficult to be removed from the adsorbent with the eluent. Therefore, 10 °C was the optimum desorption temperature for CLIT.

Furthermore, the desorption time was varied in the range of 5–30 min, with one desorption at 10 °C. As shown in Figure 7B, the desorption rate of La^3+^ increased with the increase of desorption time. When the desorption time was 25 min, the desorption rate of CLIT tended to equilibrate at around 75.07%. In addition, the desorption rate had no significant change, while the desorption continued. At a too short time, the hydrogel did not swell sufficiently, thus resulting in insufficient removal of La^3+^ ions. Once the hydrogel had swollen sufficiently at a longer time, the structure of the gel would not change further, and resulted in the desorption equilibrium.

The above desorption performance expressed that one operation was not sufficient to desorb all of the La^3+^ ions. Therefore, the desorption operation was repeated. The operation times (one to five times) were investigated and each operation was carried out at 10 °C for 25 min. As shown in Figure 7C, the desorption rate of La^3+^ from CLIT increased as the repeated times of desorption increased. When the operation times was 4, the desorption rate reached 96.72% and had no increase when the operation time increased to 5, indicating that the equilibrium had been achieved after four desorptions. The adsorption of La^3+^ by CLIT was a non-uniform adsorption on multiple molecular layers because the adsorption relationship was more fit for the Freundlich model. The single desorption process could only remove the La^3+^ adsorbed in the surface layer. During multiple operations, the La^3+^ ions adsorbed inside the CLIT could be gradually removed after the ions inside the hydrogel diffused outside the adsorbent. Therefore, multiple desorption was required to remove more La^3+^ ions from the CLIT.

### 2.6. Regeneration Performance of CLIT

The CLIT hydrogel (0.1 g dry weight) was used to adsorb and then desorb La^3+^ ions at its corresponding optimum conditions. The recycled CLIT hydrogel was used for the next adsorption. The adsorption-desorption operation was repeated 10 times to measure the regeneration performance of CLIT. The experimental results are given in Figure 7D. Both the adsorption capacity (Q_e_) and desorption rate (R%) of CLIT decreased slightly as the reused number increased. After 10 cycles of adsorption-desorption, the adsorption amount of La^3+^ by CLIT decreased from 112.21 mg/g to 84.33 mg/g. At the same time, the desorption rate reduced from 96.72% to 82.99%. The fact that a small amount of La^3+^ remained in the material after each desorption reduced the effective adsorption sites on CLIT, therefore the adsorption amount decreased gradually in the next cycle. Table 4 shows the adsorption capacity and regeneration performance of some La^3+^-imprinted adsorbents from the literature [21,22,53,54,55,56]. Compared with those reported materials, CLIT exhibits good adsorption capacity and excellent reusability. Notably, CLIT is currently the only reported rare earth metal ions-imprinted adsorbent which can be recycled by simply rinsing it with low temperature water. The generation performance indicates that CLIT has good prospects for actual application in La^3+^ recovery from solution.

## 3. Materials and Methods

### 3.1. Materials

Chitosan (CS) (Mw: 5 × 10^5^ g/mol, degree of deacetylation: 95%), N, N-methylenebisacrylamide (BIS), arsenazo III and lanthanum nitrate hexahydrate were purchased from Aladdin Biochemical Technology Co., Ltd. (Shanghai, China). NIPAM was purchased from Tokyo Chemical Industry Co., Ltd. (TCI, Tokyo, Japan). Sodium hydroxide was purchased from Silong Science Co. Ammonium persulfate (APS) and nitric acid were purchased from Guangzhou Chemical Reagent Factory (Guangzhou, China). Copper nitrate, iron nitrate nonahydrate, and aluminum nitrate nonahydrate were purchased from Shanghai Macklin Biochemical Technology Co. (Shanghai, China). All the reagents were used as received.

### 3.2. Preparation of CLIT

CLIT was prepared using the GHIP method [42] as follows: 1.0 g CS, 0.1547 g BIS, 4.2469 g APS, 2.8079 g NIPAM (nAGU of CS: nNIPAM = 1:4), 100 mL of deionized water and 1 mL of 1 mg/mL La^3+^ solution were put into a 500 mL three-neck flask. The flask was placed in a magnetic stirring water bath and stirred at 30 °C for 3 h. The temperature was then increased to 40 °C, 50 °C, 60 °C at hourly intervals and finally to 70 °C for 3 h. After the reaction, the sample was washed three times with 10 °C deionized water to remove the imprinted La^3+^ ions. CLIT was then collected after filtration and stored at 10 °C for further characterization and property investigation.

### 3.3. Characterizations

The phase transition behavior and the thermal stability of the samples were measured by a differential scanning calorimeter (DSC, Q100, TA instruments). The measurement was performed three times while repeatedly heating-cooling in the range of 10–90 °C. The DSC measurements were carried out under nitrogen protection with a ramp-up rate of 10 °C/min. The FTIR spectra of samples were measured by a Fourier infrared spectroscope (TENSOR 27, Bruker Corporation, Billerica, MA, USA) using the KBr pellet technique in the range of 500–4000 cm^−1^. Freeze-dried CLIT and CLIT-La were adhered to a carbon tape and coated with gold by a sputter coater. Their surface morphologies were then observed with a scanning electron microscope (SEM, S-3000N, Hitachi, Tokyo, Japan) at a voltage of 10 kV. Raman spectra were measured in the range of 500–4000 cm^−1^ on a spectrometer (DXRxi, Thermo Fisher, Waltham, MA, USA). The XPS spectra of CLIT and CLIT-La were measured in the range of 0–1300 eV (ESCALAB 250Xi, Thermo Fisher). The UV-Vis spectra of the samples were measured using a UV-Visible spectrophotometer (Lambda 750s, PE instruments, Santa Ana, CA, USA).

### 3.4. La^3+^ Adsorption Performance and Mechanism of CLIT

The adsorption performance of CLIT under different adsorption temperature, adsorption time, initial solution pH and initial La^3+^ concentration was studied to obtain the optimum adsorption conditions for CLIT. The experiments were carried out as follows: the CLIT hydrogel whose dry weight was 0.1 g was put into a 50 mL beaker. 20 mL solution with different La^3+^ concentration was added into the beaker, and then the beaker was placed in a shaker at a constant temperature for a period of adsorption time. The remaining La^3+^ concentration in the filtrate was measured by UV spectrophotometer through the arsenazo III method at 652 nm [57]. The amount of La^3+^ adsorbed by CLIT, Q (mg/g) was calculated according to Equation (1).

(1) $$Q=\frac{(C_{0}{\;-\;C}_{R})\times V}{W}$$

C_0_ is the initial La^3+^ concentration in the adsorption solution (mg/mL); C_R_ is the remaining La^3+^ concentration in the filtrate after adsorption (mg/mL); V is the volume of the solution (mL) and W is the dry weight of the CLIT used for adsorption (g).

The amount of La^3+^ adsorbed by CLIT was calculated and fitted by the quasi-first-order kinetic equation (Equation (2)) and quasi-second-order kinetic equation (Equation (3)).

(2) $${\ln(Q}_{e}{-Q}_{t})={\mathrm{lnQ}}_{e}{-K}_{1}t$$

(3) $$\frac{Q_{e}}{Q_{t}}=\frac{1}{K_{2}\times Q_{e}}+t$$

Q_e_ is the equilibrium adsorption amount of CLIT (mg/g); K_1_ is the primary adsorption rate constant (min^−1^); Q_t_ is the adsorption amount at time t (mg/g); and K_2_ is the secondary adsorption rate constant (g/(mg·min)).

The relationship between adsorption capacity and equilibrium concentration in the adsorption process of CLIT was described using the Langmuir and Freundlich isothermal models, shown in Equations (4) and (5).

(4) $$\frac{C_{e}}{Q_{e}}=\frac{1}{K_{L}\times Q_{m}}+\frac{C_{e}}{Q_{m}}$$

(5) $${\mathrm{lnQ}}_{e}={\mathrm{lnK}}_{F}+\frac{1}{n}\ln C_{e}$$

Q_e_ is the adsorption capacity when CLIT reaches adsorption equilibrium (mg/g); Q_m_ is the maximum theoretical adsorption capacity (mg/g); C_e_ is the La^3+^ concentration in solution when the adsorption reaches equilibrium (g/L); K_L_ (L/mg), and K_F_ is the adsorption equilibrium constant of Langmuir and Freundlich, respectively.

The selective adsorption performance of CLIT was investigated by performing the adsorption in a solution containing six kinds of metal ions (including La^3+^, Y^3+^, Gd^3+^, Al^3+^, Fe^3+^ and Cu^2+^). The concentration of all the metal ions was set at 1.9 mg/mL. The adsorption was carried out at pH 5 and 45 °C for 60 min. The concentration of each ion in the solution after adsorption was determined by ICP-OES. The adsorption capacity was calculated according to Equation (1) for each metal ion. At the same time, the hydrogel without the template La^3+^ (NIPL) was also prepared according to the procedure of CLIT described in Section 3.2. The selective adsorption performance of NIPL was also tested with the same procedure of CLIT. The distribution coefficient (D) and selective adsorption coefficients (k) of CLIT and NIPL were measured by Equations (6) and (7).

(6) $$\mathrm{DC}=\frac{{[(C}_{i}-C_{e}{)/C}_{e}]\times V}{W}$$

(7) $$k=\frac{D_{{\mathrm{La}}^{3+}}}{D_{M^{n+}}}$$

C_i_ (mg/mL) is the initial concentration of metal ions; C_e_ (mg/mL) is the equilibrium concentration of metal ions; V (mL) is the aqueous solution volume; W (g) is the dry weight of hydrogel adsorbent; D_La3+_ is the distribution coefficient of La^3+^; D_Mn+_ is the distribution coefficient of the other co-existing metal ions in the solution.

### 3.5. Desorption and Reusability of CLIT

The La^3+^-adsorbed hydrogel was desorbed and recycled by washing with cold water. The effects of temperature of washing water, desorption time, and repeated operation times of desorption on the desorption rate of CLIT were investigated. The experimental procedure was as follows: after adsorption under the optimal adsorption conditions according to the procedure described in Section 3.3, the La^3+^-adsorbed CLIT was separated from the adsorption solution using a 0.22 μm filter membrane and put into 20 mL of deionized water at a certain temperature (5–25 °C) for a certain period of time (5–30 min), which was repeated for a certain number of times (one to five times). The La^3+^ concentration in the solution was then tested after filtration. The desorption rate (R%) of CLIT was calculated according to Equation (8).

(8) $$R\%=\frac{C_{R}\times V_{R}}{Q\times W}\times 100\%$$

C_R_ is the concentration of La^3+^ in the solution (mg/mL), V_R_ is the total volume of the washing water used for desorption (mL); Q is the amount of La^3+^ adsorbed by CLIT (mg/g); and W is the dry weight of CLIT (g).

The desorbed CLIT at the optimum conditions was then used for the next adsorption cycle. The adsorption and desorption operation were carried out repeatedly for ten times at the optimum adsorption and desorption conditions to investigate the reusability of CLIT.

## 4. Conclusions

In this paper, a chitosan-based La^3+^-imprinted temperature-sensitive material (CLIT) was successfully prepared with NIPAM as the thermally responsive monomer in the presence of template La^3+^ ions. CLIT exhibited reversible thermosensitivity in the range of 28–38 °C with repeated heating and cooling. The adsorption and desorption conditions of CLIT were optimized. The adsorption amount of CLIT to La^3+^ at optimal conditions was 112.21 mg/g. CLIT expressed much better adsorption selectivity to La^3+^ than the non-imprinted NIPL, verifying the presence of the imprinting sites of La^3+^. CLIT could be well regenerated simply by rinsing with deionized water at 10 °C for 25 min after 4 repeatedly operation, with a desorption rate of 96.72%. After 10 cycles of the adsorption-desorption process, the adsorption amount to La^3+^ remained at 84.33 mg/g and the desorption rate at 82.99%. In summary, CLIT is a smart, water-recyclable, green, and selective adsorbent for La^3+^ ions which has good application potential in rare earth metal ions accumulation.

## Figures and Tables

**Table 1:** XPS binding energy of CLIT and CLIT-La.

Samples	N1s (eV)		O1s (eV)		La3d^3/2^ (eV)		La3d^5/2^ (eV)	
	C-N	N-H	C-O, COO^−^	C=O				
CLIT	400.1	399.2	532.4	531.1	-		-	
CLIT-La	401.5	399.7	533.4	531.4	852.4	856.8	835.5	840.1

**Table 2:** Selective adsorption coefficients k of CLIT and NIPL in solution containing six ions (containing Al^3+^, Cu^2+^, Fe^3+^, Gd^3+^, La^3+^ and Y^3+^).

Samples	k_La3+/Al3+_	k_La3+/Cu2+_	k_La3+/Fe3+_	k_La3+/Gd3+_	k_La3+/Y3+_
CLIT	3.61	5.66	3.09	2.27	1.83
NIPL	0.99	1.42	1.08	0.91	0.80

**Table 3:** Fitting parameters of adsorption isotherm models for CLIT.

Sample	Freundlich			Langmuir		
	Kf (L/mg)	*n*	R_f_^2^	K_l_ (L/mg)	Q_m_ (mg/g)	R_l_^2^
CLIT	2.0662	1.4914	0.9955	0.7713	130.01	0.9118

**Table 4:** Adsorption capacity and regeneration performance of La^3+^ ion-imprinted adsorbents.

La^3+^ Ion-Imprinted Adsorbent	Adsorption Capacity (mg/g)	Regeneration Times	Regeneration Efficiency	Eluents	Ref.
La-IIP-Schiff	25.0	10 cycles	About 92%	HCl	[21]
La-IIP-Azo	24.3				
La (III)-IIP-PEI/MCM-41	212.69	Not reported	Not reported	HCl	[53]
La-IIP/MCM-41	272.2	5 cycles	About 81%	HCl	[54]
La-IIP-MAA/Fe_3_O_4_-GO	110.98	5 cycles	About 90%	HCl	[22]
La-IIP	62.8	5 cycles	About 100%	HCl	[56]
CLIT	112.21	10 cycles	About 83%	deionized water	This work

## Data Availability

Not applicable.

## Supplementary Materials

The following supporting information can be downloaded at: https://www.mdpi.com/article/10.3390/ijms231810542/s1.
