# Supplementary material for: Water-Recyclable Chitosan-Based Ion-Imprinted Thermoresponsive Hydrogel for Rare Earth Metal Ions Accumulation
Source: Int J Mol Sci. 2022 Sep 11;23(18):10542. doi: 10.3390/ijms231810542 (PMC9505209; doi:10.3390/ijms231810542)
Supplement: Supplementary file 1 [file ijms-23-10542-s001.zip › ijms-1854265-supplementary.pdf]

Supporting Information

Water-recyclable chitosan-based ion-imprinted thermoresponsive hydrogel for rare earth metal ions accumulation

Yuheng Qiu[a]†, Kaiqi Ding[a]†, Liwen Tang[a], Ziyu Qin[b]*, Mengting Li[a], Xueqiong Yin[a]*

[a] Hainan Provincial Fine Chemical Engineering Research Center, Hainan University, Haikou, Hainan, 570228, P.R. China

[b]College of Chemical Engineering and Technology, Hainan University, Renmin Avenue 58th, Haikou, Hainan, China.

†Authors contributed equally to the work.

*Corresponding Author: ziyuqin@hainanu.edu.cn (Ziyu Qin); yxq88@hotmail.com (Xueqiong Yin)

Table S1 Preparation conditions of CLIT with different dosages of NIPAM and La^{3+}

Sample number	n_{CS}：n_{NIPAM}	La^{3+} dosage（mg）
CLIT1	1：3	1
CLIT2	1：4	1
CLIT3	1：5	1
CLIT4	1：3	2
CLIT5	1：4	2

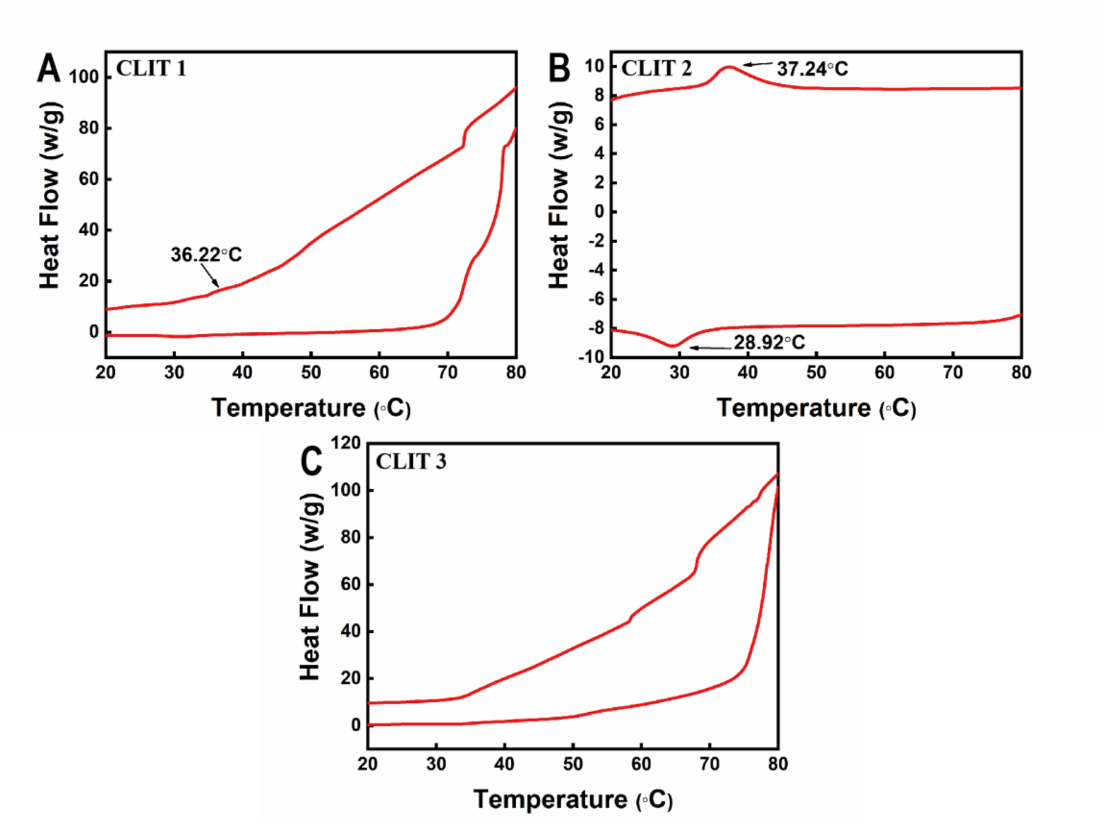

Figure S1. Effect of NIPAM dosage on thermo-sensitivity of CLIT

In order to explore the relationship between the dosage of NIPAM and the thermal response properties of the material, the dosage of the reagents was fixed at 1 g CS, 0.1547 g BIS, 4.2469 g APS and 1 mg La^{3+}, respectively. Three adsorbents with different amounts of NIPAM were prepared, including CLIT1 (n_{CS}: n_{NIPAM}=1:3), CLIT2 (n_{CS}: n_{NIPAM}=1:4), and CLIT3 (n_{CS}: n_{NIPAM}=1:5). The preparation conditions were listed in Table S1. The temperature sensitivity of CLIT1, CLIT2, and CLIT3 was determined by differential scanning calorimetry (DSC). The DSC results were shown in Figure S1.

As shown in Figure S1, during the heating process, CLIT1 (Figure S1(A)) appeared an endothermic peak at 36.22°C, but did not have obvious exothermic peak during cooling.

This may be due to NIPAM amount is insufficient to achieve reversible thermal responsiveness structure of the material. It could be seen from Figure S1(B) that when the amount of NIPAM was n_{CS}: n_{NIPAM}=1: 4, CLIT2 had an endothermic peak and an exothermic peak at 37.24°C and 28.92°C during the heating and cooling process, respectively. Which indicates that CLIT2 undergoes a reversible phase transition and exhibits good reversible thermal response properties. When the amount of NIPAM was n_{CS}: n_{NIPAM}=1: 5, CLIT3 did not show corresponding endothermic peaks and exothermic peaks during the heating and cooling processes. When NIPAM is excessive, over self-polymerization and self-aggregation of NIPAM occur. Then the graft-polymerization and crosslinking of PNIPAM with chitosan is not sufficient to form reversible structure. Therefore, in the follow-up research, the ratio of NIPAM to CS 1:4 was used.

Figure S2. The influence of La^{3+} dosage on the temperature sensitivity of CLIT

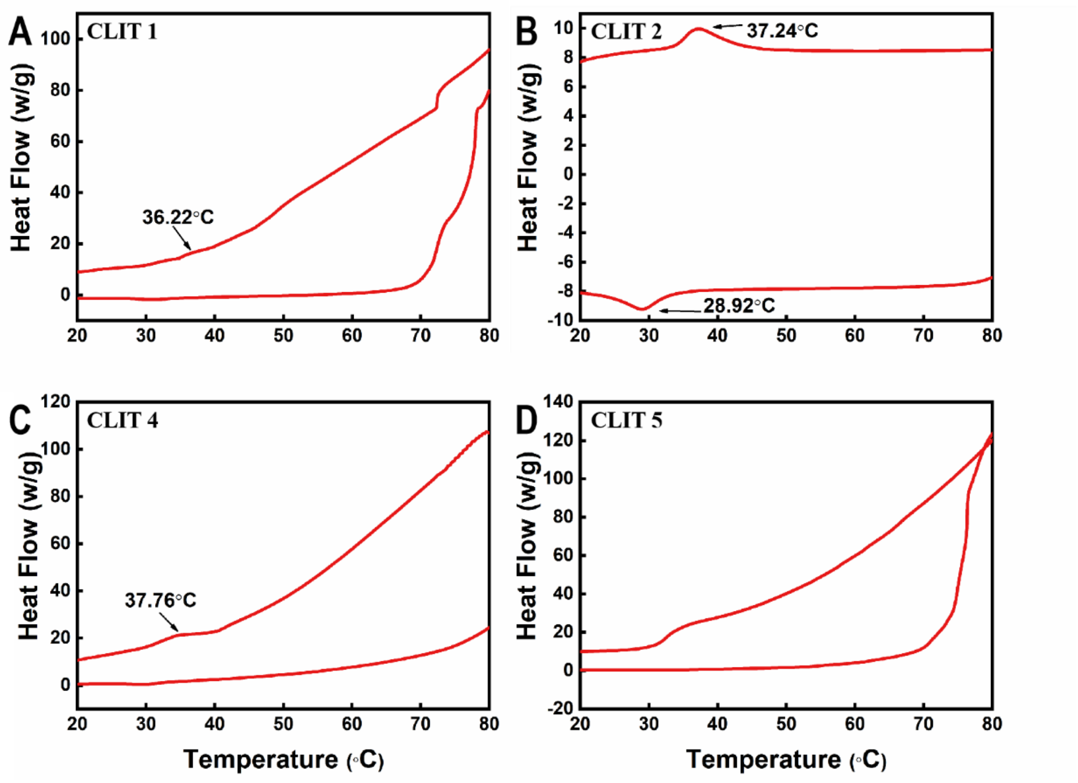

After excluding CLIT3 which was not thermally responsive, we investigated the effects of La^{3+} dosage on the thermosensitivity, with 1 mg (CLIT1, CLIT2) and 2 mg (CLIT4, CLIT5) of La^{3+} template ions. The preparation conditions were listed Table S1. The DSC test results were shown in Figure S2.

As shown in Figure S2(A, B), when the amount of NIPAM was fixed at n_{CS}: n_{NIPAM}=1:3, both CLIT1 and CLIT4 only showed an endothermic peak during the heating process, but no corresponding exothermic peak appeared during the cooling process. When the amount of NIPAM was fixed at n_{CS}: n_{NIPAM}=1:4, only CLIT2 prepared with 1 mg La^{3+} ions exhibited a reversible phase transition. Therefore, the optimal preparation conditions of CLIT are

n_{CS}: n_{NIPAM}=1:4, and the dosage of La^{3+} being 1 mg.
